# Primary Gastric Hodgkin Lymphoma: A Case Report

**DOI:** 10.34172/mejdd.2025.448

**Published:** 2025-10-31

**Authors:** Hana Ben Hammamia, Dorra Jabr, Raoudha Mansouri, Malek Sayadi, Karima Kacem, Raihane Ben Lakhal, Ghada Sahraoui, Karima Mrad

**Affiliations:** ^1^Clinical Hematology Department in Aziza Othmana Hospital, the Faculty of Medicine of Tunis, Tunis EL Manar University, Tunisia; ^2^Anatomopathology Department in Salah Azaiez Institute, the Faculty of Medicine of Tunis, Tunis EL Manar University, Tunisia

**Keywords:** Classical Hodgkin lymphoma, Gastrointestinal Hodgkin lymphoma, Extranodal involvement

## Abstract

Hodgkin lymphoma is among the most frequent hematologic malignancies that affects the lymphatic system. Primary extranodal involvement in Hodgkin disease is unusual. We report the case of primary gastric Hodgkin lymphoma in a 50-year-old man, who complained about epigastralgia and poor appetite. An upper gastrointestinal endoscopy, biopsy, and immunohistochemical exam confirmed the diagnosis of Hodgkin lymphoma. Primary gastric Hodgkin lymphoma is a very rare entity; clinicians and anatomopathologists should be sensitized to its uncommon extranodal localization.

## Introduction

 Hodgkin lymphoma is the most frequent lymphoproliferative neoplasm. Lymph nodes are mainly the origin of this disease. However, sometimes Hodgkin disease can arise from extra nodal tissues.^1^

 Initial staging with the modified Ann-Arbor classification system is crucial to distinguish between stage IV Hodgkin disease and contiguous (E-stage) disease, which will affect the treatment decisions. The designation E for extranodal disease is not available for patients with advanced-staged disease.^2^

 Most published studies on Hodgkin lymphoma of primary extranodal involvement are case reports.^1^

 The purpose of our study is to describe an unusual case of isolated primary gastric Hodgkin disease.

## Case Report

 We report the case of a 50-year-old male with a 6-month history of poor appetite, epigastralgia, weight loss, and body weakness. His medical history was significant of pernicious anaemia treated by vitamin B12. The family and social history were unremarkable.

 Clinical examination showed an epigastric tenderness with no enlargement of the liver or spleen or ascites. There was no peripheral lymphadenopathy.

**Figure 1:**
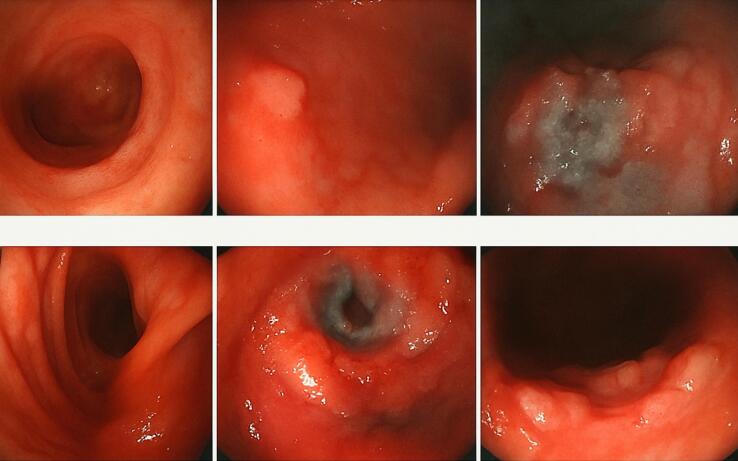
Endoscopic photos of the patient

**Figure 2:**
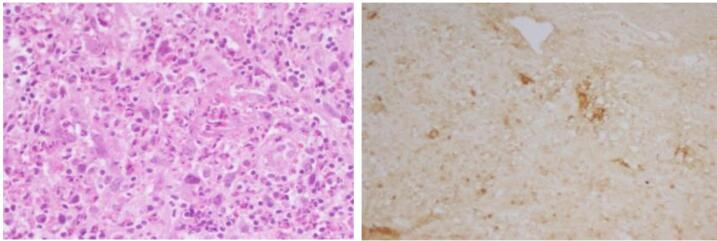
Histopathological pictures and immunophenotype CD 30^+^

 An upper gastrointestinal endoscopy (Figure 1) revealed a submucosal nodule of the stomach and duodenal ulceration. Histological examination of gastric and duodenal biopsies revealed an antropyloric mucosa occupied by large Reed-Sternberg cells mixed with lympho-plasmocytic polymorphic elements. These cells were CD30^+^, CD15^–^, CD20^–^, Fascine^+^, and Pax 5^+^, consistent with classical Hodgkin lymphoma (Figure 2). Duodenal ulceration histology did not show signs of malignancy. Diagnosis of gastric Hodgkin lymphoma was retained.

**Figure 3:**
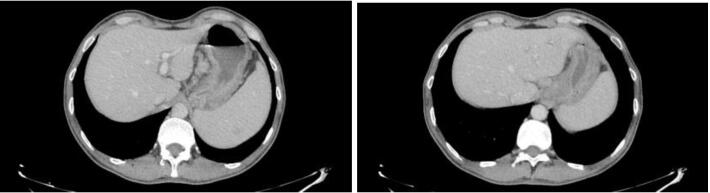
Scan features showing gastric thickening

 Chest radiography showed no mediastinal enlargement. Cervical, thoracic, abdominal, and pelvic scans showed multiple celiac, mesenteric, peri-duodenal, lumbo-aortic, and right primitive iliac adenopathy, gastric thickening, and moderate splenomegaly measuring 15 cm long axis (Figure 3).

**Figure 4:**
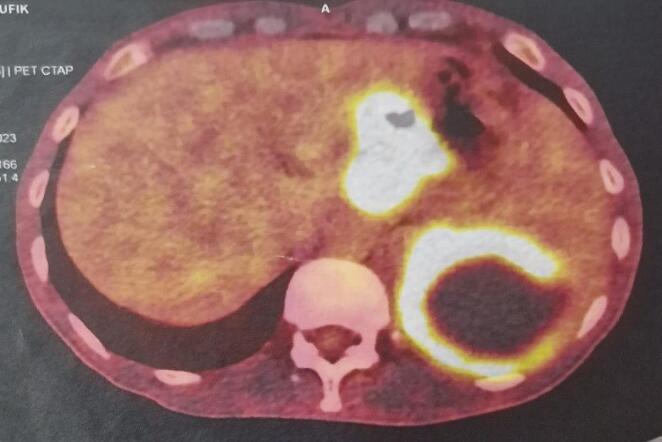
Positron emission tomography showing a gastric hypermetabolism

 Positron emission tomography (PET) showed irregular thickening of the small hypermetabolic gastric curvature associated with hypermetabolic peri gastric lymph node foci, hypermetabolic lymphadenopathy of the splenic hilum, latero-aortic hypermetabolic lymphadenopathy, and left colic focal hypermetabolism (Figure 4).

 Cell Blood count showed WBC at 15000/mm^3^, lymphocytes at 1350/mm^3^, and hemoglobin (Hb) at 12.7 g/dL. Sedimentation rate was 90 mm. Liver enzymes and other relevant biochemistry tests were normal.

 The patient, staged as IVBb gastric disease, received two lines of chemotherapy, but has not responded to treatment. He is currently awaiting the third line of treatment.

## Discussion

 Hodgkin lymphoma is the most diagnosed cancer in young adults aged from 15 to 19 years. A literature review made by Punnett and colleagues showed a prevalence of 10-12% in the paediatric population under the age of 14 years and a prevalence of 35% over the age of 55 years.^3^ The gastrointestinal tract, especially the stomach, is frequently involved in non-Hodgkin lymphoma; however, it is rare in Hodgkin lymphoma.^4^ Previous studies showed that the frequency of primary Hodgkin gastric lymphoma is less than 1% of all gastric lymphomas.^5^

 The particularity of the clinical presentation in our case is the initial and isolated gastric involvement. The literature review identified only 25 cases of Hodgkin’s lymphoma of the stomach.^6^

 A set of criteria had been established by Dawson and others, including the absence of palpable adenopathy when the patient was first seen, the absence of mediastinal enlargement, normal white blood cell count, the bowel lesion’s predominance at laparotomy with affected lymph nodes only in its immediate neighborhood, and the absence of lymphomatous involvement of spleen and liver involvement.^7^ In our case, almost all these criteria were present.

 The diagnosis of primary Hodgkin disease is challenging. Gastrointestinal endoscopy revealed non-specific gastric ulceration. Moreover, the presence of Reed-Sternberg cells in histological diagnosis can be seen in many stomach malignancies, including Reed-Sternberg-like cells, such as those found in histiocytosis, anaplastic large cell lymphomas, and peripheral T-cell lymphomas.^4^

 In addition to conventional histological examination, immunohistochemical staining is crucial to differentiate between Hodgkin and non-Hodgkin lymphoma and confirm the diagnosis. The panel immunophenotyping used to study Hodgkin lymphoma is based on CD3, CD20, CD30, CD 15 and PAX5.^7^ Classical Hodgkin lymphoma (CHL) expresses the positivity of CD30 and CD15 and the negativity of B-cell markers: CD20. In our present case, our patient showed the expression of CD30^+^, pax5^+^, and CD15^-^, which is consistent with the literature, as in some CHL cases, CD15 expression is completely absent. The expression of CD15 varies between individuals, and even in the same individual, it varies in cellular distribution, focus, and intensity. Pax 5 confirms the B-cell nature of Hodgkin’s Reed-Sternberg cells, and it is expressed in the majority of CHL.^8^

## Conclusion

 Hodgkin disease of the stomach is extremely rare. We reported this case to insist on the importance of histological examination and immunohistochemical staining.
